# Optimization of the VSV-G backbone for amino terminal fusion with nanobodies allowing its specific retargeting to HER2 receptors

**DOI:** 10.1016/j.omton.2025.201065

**Published:** 2025-09-25

**Authors:** Isoline Duquénois, Stéphanie Thébault, Sarah Johari, Hélène Raux, Malika Ouldali, Sandrine Moutel, Cécile Lagaudrière-Gesbert, Franck Perez, Aurélie A. Albertini, Yves Gaudin

**Affiliations:** 1 Institute for Integrative Biology of the Cell (I2BC), CEA, CNRS, Université Paris-Saclay, 91198 Gif-sur-Yvette, France; 2 Recombinant Antibody Platform, Institut Curie, PSL Research University, Sorbonne Université, Centre National de la Recherche Scientifique, UMR 144, 75005 Paris, France; 3 Dynamics of Intracellular Organization Laboratory, Institut Curie, PSL Research University, Sorbonne Université, Centre National de la Recherche Scientifique, UMR 144, 75005 Paris, France

**Keywords:** MT: Regular Issue, vesicular stomatitis virus, lentivirus, VSV glycoprotein, nanobodies, pseudotype, oncovirotherapy, HER2, retargeting, experimental evolution

## Abstract

Vesicular stomatitis virus (VSV) is a promising oncolytic virus. Additionally, its glycoprotein G (VSV-G) is the most commonly used envelope glycoprotein to pseudotype lentiviral vectors for gene therapy. However, VSV receptors (low-density lipoprotein receptor [LDL-R] family members) are ubiquitous and expressed at the surface of non-target cells, precluding *in vivo* gene therapy. Recently, we identified VSV-G mutants that no longer bind to LDL-R but retain their fusion activity. This created the opportunity to specifically retarget the glycoprotein to the desired receptors. Here, we engineered chimeric glycoproteins by fusing nanobodies to the amino terminus of VSV-G. Using experimental evolution, we identified two mutations in VSV-G improving the folding and functionality of chimeric glycoproteins, regardless of the nanobody inserted at the amino terminus. We then generated chimeric glycoproteins using several nanobodies targeting HER2 receptor, and into these chimeras, we introduced additional mutations that abolish recognition of LDL-R. VSV and lentiviruses pseudotyped with these glycoproteins specifically infect cells expressing HER2. We have, therefore, identified VSV-G mutations that optimize its backbone to tolerate amino-terminal insertion of a nanobody and establish proof of concept that this approach can be used to confer a new tropism on VSV-G. This paves the way for the development of targeted *in vivo* therapies.

## Graphical abstract

**Figure:**
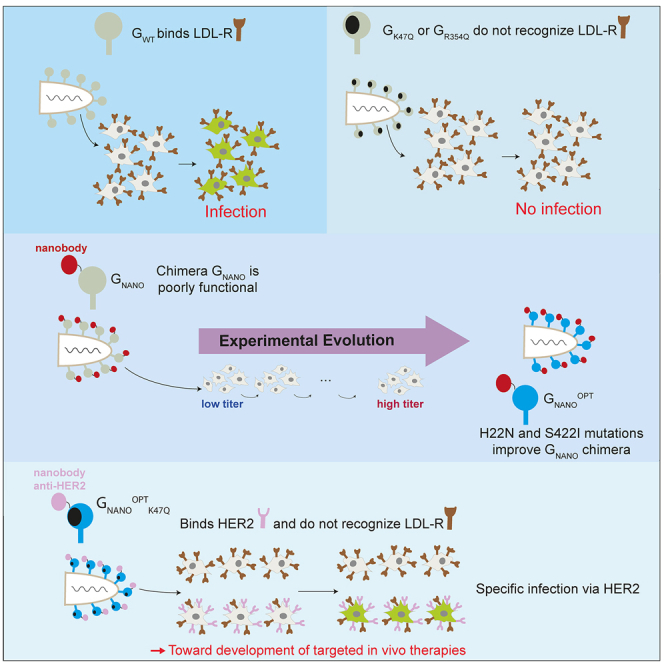

## Teaser

Duquénois and colleagues engineered vesicular stomatitis virus (VSV) glycoproteins fused to nanobodies and identified backbone mutations enhancing their folding and function. Combined with mutations abolishing low-density lipoprotein receptor (LDL-R) binding, these chimeras specifically retarget VSV and lentiviral vectors to HER2-expressing cells. This work establishes proof-of-concept for tunable tropism, paving the way for targeted *in vivo* therapy.

## Introduction

Vesicular stomatitis virus (VSV) is a promising oncolytic virus particularly for the treatment of refractory solid tumors.^1^^,^^2^^,^^3^^,^^4^^,^^5^^,^^6^ Indeed, it replicates preferentially in tumor cells due to their interferon (IFN) pathway defects, and the subsequent lysis of infected cells induces the release of tumor antigens, which then stimulate the anti-tumor immune response.^2^^,^^7^ Its glycoprotein G (VSV-G) is also the most commonly used envelope glycoprotein to pseudotype lentiviral vectors for gene therapy approaches.^8^^,^^9^ In both cases, the lack of specificity of VSV-G limits the spectrum of its use. Indeed, VSV-G binds to VSV receptors, which are the low-density lipoprotein receptor (LDL-R) and other members of its family.^10^ These receptors are present on the surface of almost all cell types. In virotherapy, this can result in off-target toxicity, potentially neurotoxicity.^11^^,^^12^^,^^13^ This lack of specificity also precludes *in vivo* gene therapy (i.e., by administering the lentiviral vector to a specific organ or area in the body). There is, therefore, a strong need to develop glycoproteins derived from VSV-G specifically targeting cells of interest for therapeutic purposes.

VSV-G has two functions essential for viral entry into the host cell.^14^ First, it initiates infection by binding to a cellular receptor (the LDL-R or a member of its family),^10^ which triggers endocytosis of the virion.^15^^,^^16^ Then, the acidic environment of the endosome induces a huge conformational change of VSV-G from its pre-fusion state to its post-fusion state, which catalyzes the fusion of the viral envelope with the endosomal membrane.^14^^,^^17^^,^^18^^,^^19^ This results in the release of the viral ribonucleoprotein in the cytoplasm for subsequent stages of infection.

**Figure 1:**
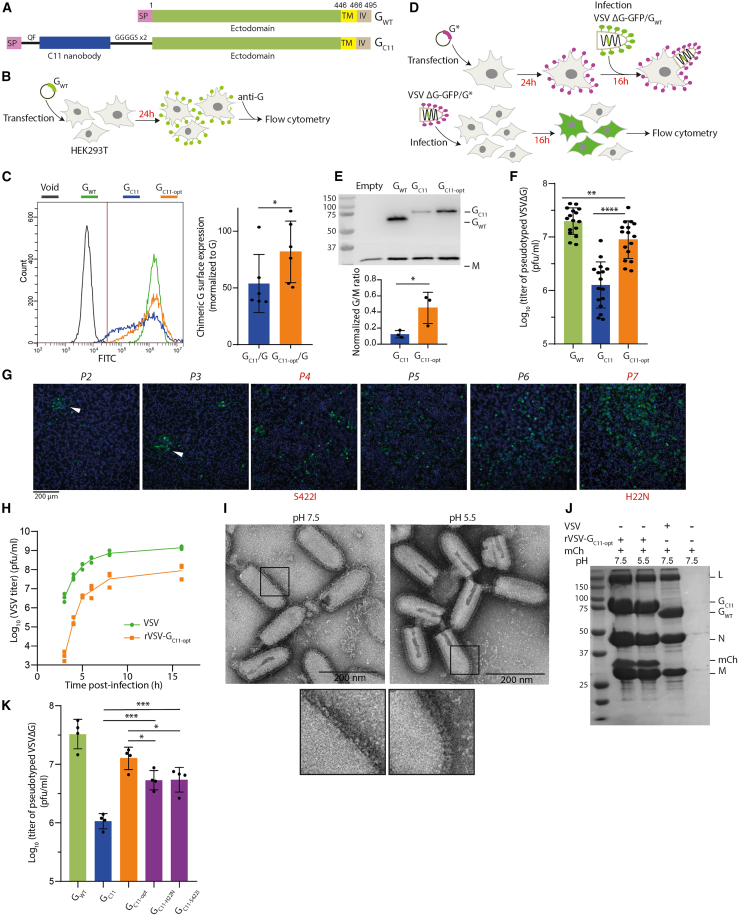
Optimization of the VSV-G backbone to tolerate an amino-terminal fusion with the C11 nanobody (A) Schematic representation of G_WT_ and the G_C11_ chimera. QF: inserted dipeptide; GGGGS x 2: flexible linker; TM: transmembrane domain; IV: intraviral domain. (B) Schematic of the experimental design to evaluate transport of VSV-G and its variants to the cell surface. (C) Left: Transport of G_WT_, G_C11_, and G_C11-opt_ to the cell surface of transfected HEK293T cells. G was detected on the surface of non-permeabilized cells using 8G5F11 mAb specific for VSV-G ectodomain and a secondary antibody conjugated to Alexa Fluor 488. Cells were analyzed by flow cytometry. Right: Median surface expression levels, measured using flow cytometry, of G_C11_ and G_C11-opt_ normalized to median concentration of G_WT_ on the surface of HEK293T cells. (D) Schematic of the experimental design to generate VSVΔG-GFP pseudotyped by G chimeras (top) and to measure their infectivity (bottom). (E) Incorporation of G_WT_, G_C11_, and G_C11-opt_ into VSVΔG-GFP viral particles assessed by western blot analysis using an anti-VSV-G and an anti-VSV M antibody. In the bar diagram, the G/M ratio in VSVΔG-GFP pseudotyped by G_C11_ and G_C11-opt_ was normalized to that in VSVΔG-GFP pseudotyped by G_WT_. (F) Infectivity of VSVΔG-GFP pseudotyped with G_WT_, G_C11_, and G_C11-opt_ in HEK293T cells. VSVΔG-GFP viruses pseudotyped with WT or chimeric glycoproteins produced in the same conditions (see material and methods) were used to infect HEK293T cells during 16 h. The percentage of infected cells was measured by counting GFP-expressing cells by flow cytometry and used to calculate the viral titer. (G) Experimental evolution of rVSV-G_C11_ recombinant virus in BSR cells. rVSV-G_C11_ was serially passaged in BSR cells, and infected cells were labeled using an anti-N antibody and a goat anti-mouse IgG secondary antibody conjugated to Alexa Fluor 488. DAPI was used to stain nuclei. The S422I (after P4) and H22N (after P7) mutations invaded the viral population. Arrowheads indicate foci of infected cells. (H) Growth curves of VSV WT and rVSV-G_C11-opt_. At each time point, the viral titer was measured using plaque assay. (I) Negative staining electron microcopy of rVSV-G_C11-opt_ virions incubated at pH 7.5 and 5.5. Insets show the magnification of the viral membrane revealing the typical shape of VSV-G at high and low pH. (J) Binding of mCherry protein to rVSV-G_C11-opt_. Indicated viruses were incubated with mCherry at indicated pHs, centrifuged, and the pellet was analyzed by SDS-PAGE. L, N, and M indicate the positions of viral proteins; G_WT_ and G_C11-opt_, the G proteins; and mCh, the precipitated mCherry. (K) Infectivity of VSVΔG-GFP pseudotyped with G_WT_, G_C11_, G_C11-opt_, G_C11-H22N_, and G_C11-S422I_ in HEK293T cells. VSVΔG-GFP viruses pseudotyped with WT or chimeric glycoproteins, produced in the same conditions, were used to infect HEK293T cells during 16 h. The percentage of infected cells was measured by counting GFP-expressing cells by flow cytometry and used to calculate the viral titer. In (C), (E), (F), (H), and (K), data points represent replicates of at least three independent experiments. Errors bars indicate SD. In (C) and (E), statistically significant differences between G_WT_, G_C11_, and G_C11-opt_ are indicated by asterisks (∗*p* < 0.05; ∗∗*p* < 0.005; ∗∗∗*p* < 0.0005; ∗∗∗∗*p* < 0.00005).

VSV-G is a type I glycoprotein, synthesized on endoplasmic reticulum-bound ribosomes. After cleavage of its amino-terminal signal peptide (SP), the mature glycoprotein is 495-aa (amino acid) long. It is anchored in the membrane by a single α-helical transmembrane (TM) segment (Figure 1A). Most of the mass of VSV-G (residues 1-446) is located outside the viral membrane and constitutes its amino-terminal ectodomain. The structures of pre- and post-fusion trimers of VSV-G were determined revealing the details of the fusogenic conformational change.^18^^,^^19^^,^^20^ More recently, crystal structures of the pre-fusion conformation of VSV-G ectodomain in complex with two cysteine-rich (CR) domains of the LDL-R have also been determined.^21^ The binding site of the CR domains on VSV-G was the same in both crystal structures and allows the identification of two basic residues (K47 and R354), which are essential for the interaction between VSV-G and its receptor. Mutant glycoproteins in which those two residues are replaced by either an alanine or a glutamine are unable to bind LDL-R CR domains and cannot pseudotype a recombinant VSV (VSVΔG-GFP) in which the G gene is replaced by the GFP reporter gene.^21^ Nevertheless, these mutant glycoproteins retain their ability to induce syncytia formation at low pH when expressed in cells by plasmid transfection.^21^ Therefore, the fusion activity and receptor-binding activity of VSV-G can be decoupled, which paves the way for the development of VSV-G-derived glycoproteins with modified tropism.

A possible strategy to retarget VSV glycoprotein toward a receptor of interest expressed at the cell surface consists of fusing it to a protein domain specifically binding this receptor. Nanobodies (also known as VHH or single-domain antibodies) are an attractive class of specific binders.^22^^,^^23^ They are stable and rather small, and several libraries are available for screening of high-affinity bindings against a specific target.^24^^,^^25^ Thus, we constructed chimeric glycoproteins with a nanobody inserted between the SP and the ectodomain of VSV-G. However, such a fusion of a nanobody at the N terminus of VSV-G affected the properties of the glycoprotein. We, thus, carried out experimental evolution to optimize the backbone of VSV-G so that it tolerates better this insertion. Two mutations were selected in the VSV-G backbone that were shown to enhance the pseudotyping ability of several chimeras involving distinct nanobodies, several of which were directed against the receptor tyrosine-protein kinase erbB-2 (HER2, overexpressed at the surface of malignant cells^26^ and used for targeted drug delivery^27^). In these improved chimeras containing nanobodies recognizing HER2, we introduced the mutations K47Q or R354Q abolishing the recognition of LDL-R by VSV-G.^21^ These mutated chimeras efficiently pseudotype both VSVΔG-GFP and lentiviruses and specifically retarget them toward HER2-expressing cells. This work, therefore, provides an optimized VSV-G backbone that allows the construction of chimeric VSV-Gs specifically targeting cells of interest using nanobodies.

## Results

### Construction and characterization of a chimeric glycoprotein fused to a nanobody

We constructed a chimeric glycoprotein (referred as G_C11_) with a nanobody (C11, directed against mCherry)^25^ inserted between the SP and the ectodomain so that the nanobody will be located at the N-terminal end of the mature glycoprotein. The nanobody was flanked by an insertion of a QF dipeptide (to optimize the SP cleavage) and a flexible linker (GGGGSGGGGS) before VSV-G ectodomain (Figure 1A).

The choice of a nanobody recognizing mCherry was a carefully considered choice. Indeed, we then wanted to specifically improve the VSV-G backbone by experimental evolution so that it had increased tolerance to such an N-terminal insertion of a nanobody (see below) while avoiding a gain of function (GOF) at the nanobody level (e.g., mutations leading to an increased affinity for its ligand).

We transfected HEK293T cells with this construct to evaluate G_C11_ surface expression (Figure 1B). Non-permeabilized cells were labeled using the monoclonal antibody (mAb) 8G5F11, directed against VSV-G ectodomain and quantified by flow cytometry. Surface expression of G_C11_ was negatively affected compared to that of the wild-type (WT) glycoprotein (VSV-G_WT_) (Figure 1C), and the median density of G_C11_ protein present at the cell surface was 53% ± 23% of that of VSV-G_WT_ (Figure 1C).

We also assessed the ability of G_C11_ to pseudotype a recombinant VSV (VSVΔG-GFP) in which the G envelope gene was replaced by the GFP gene (Figure 1D). Chimeric glycoprotein incorporation into the envelope of the particles present in the supernatant was confirmed by western blot. Incorporation of the G_C11_ chimera into the viral membrane was less efficient than that of VSV-G_WT_ (Figure 1E). Pseudotyped viruses were then used to infect HEK293T cells, and their infectivity was analyzed by counting GFP-expressing cells using flow cytometry. The infectious titer of VSVΔG-GFP pseudotyped with G_C11_ was 1.3 log_10_ (20-fold) lower than of VSVΔG-GFP pseudotyped with VSV-G_WT_ (Figure 1F). This indicated that the fusion of the nanobody at the N terminus of VSV-G negatively affected the properties of the glycoprotein.

### Optimization of VSV-G backbone by experimental evolution

We decided to perform experimental evolution to optimize the backbone of VSV-G so that it tolerates an N-terminal fusion with a nanobody. For this, we introduced the gene encoding G_C11_ in place of that encoding G in the viral genome to construct the rVSV-G_C11_ recombinant virus. We hypothesized that selection for mutations optimizing the VSV-G backbone for insertion of the C11 nanobody would also optimize the VSV-G backbone for insertion of other nanobodies. The resulting recombinant virus only formed small infection foci in the plaque assay (Figure 1G) and had a titer of ∼10^5^ plaque-forming unit (PFU)/mL (Table 1). We serially passaged the recombinant virus to select for mutants with improved fitness. After 4 passages, mutation S422I (Table 1) had invaded the viral genome population with only minor changes in the infectious phenotype. After 7 passages, we observed a strong increase in the viral infectivity. This was due to the selection of a second mutation H22N (Table 1). No other mutation was detected after 10 passages, and we decided to stop to passage the virus (Figure 1G). Of note, mutation S422I had been selected in a different context (as a compensatory mutation of the deleterious mutation H407A) and demonstrated to stabilize the pre-fusion conformation of VSV-G.^20^

**Table 1:** Adaptation of rVSV-G_C11_ through serial passaging in BSR cells

Passage	Viral titer	Mutation	Original codon	G_C11-opt_ codon
P1	8.5 × 10^4^ PFU/mL	–	–	–
P2	1.5 × 10^5^ PFU/mL	–	–	–
P3	1.4 × 10^6^ PFU/mL	–	–	–
P4	1.1 × 10^6^ PFU/mL	S422I	AGT	ATT
P5	2.9 × 10^6^ PFU/mL	–	–	–
P6	2 × 10^6^ PFU/mL	–	–	–
P7	7 × 10^7^ PFU/mL	H22N	CAT	AAT
P8	7 × 10^7^ PFU/mL	–	–	–
P9	9 × 10^7^ PFU/mL	–	–	–

Passage number with the corresponding titer on BSR cells and the identified mutation in VSV-G ectodomain. For each mutation, the original codon and the codon in G_C11-opt_ are indicated.

### Characterization of recombinant rVSV-G_C11-opt_ and its glycoprotein

We characterized the resulting virus rVSV-G_C11-opt_ (opt for optimized indicating that VSV-G contains mutations H22N and S422I). We compared the one-step growth curve of rVSV-G_C11-opt_ with that of VSV WT. The titer of the recombinant virus after 16 h of infection in BSR cells was ∼10^8^ PFU/mL, i.e., 1 log_10_ lower than that of VSV WT (Figure 1H).

We also investigated the structure of G_C11-opt_ at the surface of the recombinant virus using electron microscopy after negative staining. At pH 7.5, both VSV-G_WT_ and G_C11-opt_ formed the characteristic ∼8-nm-wide glycoprotein layer.^17^ At pH 5.5, at the surface of the viral particles, the spikes were individualized, allowing the visualization of 12-nm-long spikes in their post-fusion conformation that stood perpendicular to the membrane (Figure 1I).^17^ This indicated that G_C11-opt_ undergoes the low pH-induced structural transition.

We then verified the ability of G_C11-opt_ at the surface of the recombinant virus to bind purified mCherry at pH 7.5 and 5.5. For this, the virus was incubated with mCherry at indicated pH followed by ultracentrifugation through a sucrose cushion. Under both pH conditions, mCherry was found associated with the recombinant virus but not with the WT virus in the pellet, indicating that G_C11-opt_ binds the mCherry via the C11 nanobody (Figure 1J). The C11 nanobody is thus properly folded and accessible in both the pre- and post-fusion states of VSV-G at the viral surface.

We also compared the surface expression of G_C11-opt_ with that of G_WT_ and G_C11_. Cells were transfected with plasmids encoding the corresponding glycoproteins, and the surface expression was quantified by flow cytometry using mAb 8G5F11. G_C11-opt_ was significantly more expressed at the cell surface than G_C11_. Indeed, the median density of G_C11-opt_ protein present at the cell surface was 81% ± 24% of that of G_WT_ (Figure 1C). Therefore, mutations H22N and S422I improve chimera transport to the cell surface.

We compared the ability of G_C11-opt_, G_WT_, and G_C11_ to pseudotype VSVΔG-GFP. Chimeric glycoprotein incorporation into the envelope of the viral particles was verified by western blot, revealing that G_C11-opt_ chimera was more efficiently incorporated than G_C11_ (Figure 1E). Pseudotyped viruses were then used to infect HEK293T cells, and their infectivity was analyzed by counting GFP-expressing cells using flow cytometry (Figure 1F). The infectious titer of the production of VSVΔG-GFP pseudotyped with G_C11-opt_ (∼10^6.95^ PFU/mL) was slightly lower than that of VSVΔG-GFP pseudotyped with G_WT_ (∼10^7.3^ PFU/mL) and ∼0.85 log_10_ (i.e., 7-fold) higher than that of the VSVΔG-GFP pseudotyped with G_C11_, confirming that the two mutations H22N and S422I optimize the infectivity of the chimera.

Finally, we investigated the effect of individual mutations H22N and S422I and compared the ability of the glycoproteins G_C11-H22N_ and G_C11-S422I_ to pseudotype VSVΔG-GFP with that of G_C11-opt_ and G_C11_. Individually each mutation improved the titer of the pseudotyped virus compared to the titer of G_C11_, however, to a lesser extent than the double mutation, indicating an additional effect of each mutation (Figure 1K).

### Hydrophobic residues at position 422 combined with Asn at position 22 optimize VSV-G for nanobody insertion

**Figure 2:**
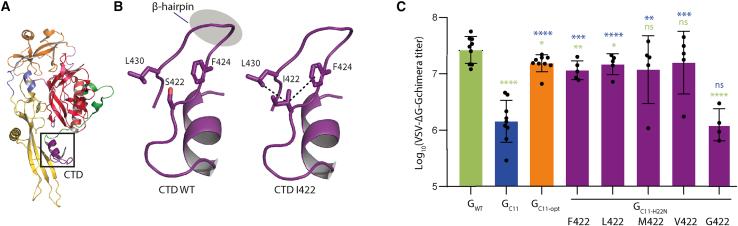
Hydrophobic residues at position 422 optimize VSV-G for nanobody insertion (A) Structural environment of residue S422 in the crystal structure of the pre-fusion conformation of VSV-G. (B) Mutation S422I stabilizes G C-terminal domain (CTD) through hydrophobic stacking with residues F424 and L430. (C) Infectivity of VSVΔG-GFP pseudotyped with G_WT_, G_C11_, G_C11-opt_, G_C11-opt-S422F_, G_C11-opt-S422L_, G_C11-opt-S422M_, G_C11-opt-S422V_, and G_C11-opt-S422G_ in HEK293T cells. VSVΔG-GFP viruses pseudotyped with G_WT_ or chimeras, produced in the same conditions, were used to infect HEK293T during 16 h. The percentage of infected cells was measured by counting GFP-expressing cells by flow cytometry and used to calculate the viral titer. Data points represent replicates of independent experiments. Error bars indicate SD. Statistically significant differences with G_C11_ (respectively G_WT_) are indicated by blue (respectively green) asterisks (∗*p* < 0.05; ∗∗*p* < 0.005; ∗∗∗*p* < 0.0005; ∗∗∗∗*p* < 0.00005; ns, not significant).

The structure of VSV-G ectodomain in its pre-fusion conformation reveals that residue S422 points toward F424 and L430. As already proposed,^20^ the selection of an isoleucine at position 422 likely stabilizes the β-hairpin structure through hydrophobic stacking with residues F424 and L430 (Figures 2A and 2B). The codon encoding S422 in the G_WT_ gene is AGU (Table 1), and the only codon encoding a hydrophobic residue that is accessible by a single-nucleotide change is AUU, coding for isoleucine. Consequently, other hydrophobic aa were not directly accessible during experimental evolution. We decided to test the impact of other hydrophobic aa residues (L, V, F, M) as well as a glycine (G) in this position. Thus, we replaced isoleucine 422 in G_C11-opt_ by those aa leading to constructs G_C11-opt-L422_, G_C11-opt-V422_, G_C11-opt-F422_, G_C11-opt-M422_, and G_C11-opt-G422_ (note that all these constructs contain the H22N mutation). We then assessed the pseudotyping efficiency of these constructs using VSVΔG-GFP. The infectious titers of VSVΔG-GFP pseudotyped with the chimeric glycoproteins G_C11-opt-L422_, G_C11-opt-V422_, G_C11-opt-F422_, or G_C11-opt-M422_ were similar to that of VSVΔG-GFP pseudotyped with G_C11-opt_, significantly higher than that of VSVΔG-GFP pseudotyped with G_C11_ (Figure 2C). On the other hand, the infectious titer of virus pseudotyped with chimeric glycoproteins G_C11-opt-G422_ was similar to that with G_C11_ (Figure 2C). This demonstrated the importance of a hydrophobic residue at position 422 for optimizing VSV-G backbone to tolerate nanobody insertion.

### The optimized G backbone is functional for insertion of other nanobodies

We investigated whether the double mutation H22N and S422I also improved the functionality of G chimeras fused with other nanobodies. For this, we inserted in the G_WT_ or G_opt_ backbones 4 other nanobodies from the same library as C11^25^: 8G5 directed against the carcinoembryonic antigen cell adhesion molecule (CEACAM), 9C2 directed against mucin 1 protein (MUC1), C8 and A10 both targeting HER2, and 6 others (H1–H6) also targeting HER2, from another library (European patent EP-3643726-A1), whose sequences were available in databanks.

The resulting constructions were called G_C8_, G_8G5_, G_A10_, G_9C2_, G_H1_ G_H2_ G_H3_ G_H4_, G_H5_, and G_H6_ when the insertion was in the backbone of G_WT_, and G_C8-opt_, G_8G5-opt_, G_A10-opt_, G_9C2-opt_, G_H1-opt_, G_H2-opt_, G_H3-opt_, G_H4-opt_, G_H5-opt_, and G_H6-opt_ when the insertion was in the optimized G backbone containing the mutations H22N and S422I.

**Figure 3:**
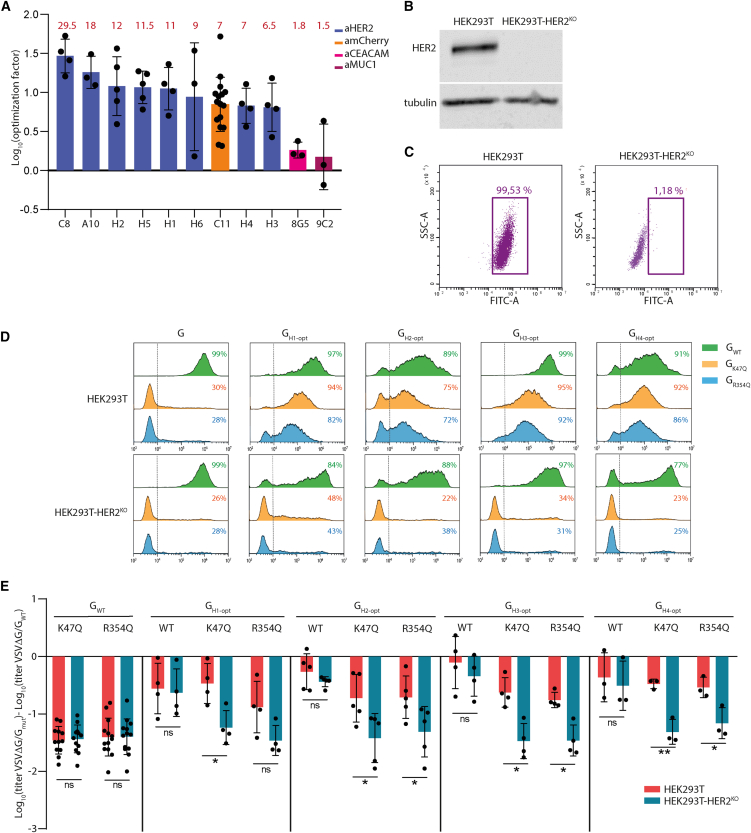
Retargeting of VSVΔG-GFP toward HER2-expressing cells (A) Optimization factor of the titer of VSVΔG-GFP pseudotyped with the chimeric G in the optimized backbone compared with VSVΔG-GFP pseudotyped with a chimera in a non-optimized backbone. VSVΔG-GFP viruses, produced in the same conditions, pseudotyped with chimeras in an optimized or non-optimized backbone were used to infect HEK293T cells during 16 h. The percentage of infected cells was measured by counting GFP-expressing cells by flow cytometry and used to calculate the viral titer. The histogram shows the mean difference between the log_10_ of the titer of VSVΔG-GFP pseudotyped with the optimized chimera and the log_10_ of the titer of VSVΔG-GFP pseudotyped with the non-optimized chimera. The factor of optimization is indicated in red for each chimera. (B) Expression of HER2 in HEK293T and HEK293T-HER2^KO^ cells. Cell lysates were analyzed by western blot using anti-HER2 and anti-tubulin antibodies. (C) Cell surface expression of HER2 in HEK293T and HEK293T-HER2^KO^ cells. Expression of HER2 was analyzed using flow cytometry with an anti-HER2 as a primary antibody and a secondary antibody conjugated to Alexa Fluor 488. The percentage of HER2-positive cells is indicated. (D) Flow cytometry graph showing the infectivity in HEK293T and HEK293T-HER2^KO^ cells of VSVΔG-GFP pseudotyped with G_WT_, G_K47Q_, G_R354Q_, or with optimized G chimeras fused to nanobodies H1–H4 (recognizing HER2) in which mutations K47Q or R354Q have been introduced or not. VSVΔG-GFP viruses pseudotyped with these glycoproteins were used to infect indicated cell lines during 16 h. The percentage of infected cells was measured by counting GFP-expressing cells by flow cytometry and is indicated on the top right of each graph. (E) Histogram showing the log_10_ of the titer of viruses pseudotyped with mutated or chimeric Gs normalized to that pseudotyped with G_WT_ in the indicated cell line. Each point corresponds to a single experiment as in (D). A value of 0 means that the titer of the virus pseudotyped with mutated or chimeric Gs is equal to the titer of the virus pseudotyped with G_WT_ (in the indicated cell line). Viral titers were calculated from the percentage of cells expressing GFP. The log_10_ of the titer of VSVΔG-GFP virus pseudotyped with G_WT_ was 7.37 ± 0.34 in HEK293T and 7.39 ± 0.38 in HEK293T-HER2^KO^ cells (*n* = 13). In (A) and (E), error bars represent the SD for experiments carried out at least in triplicate (see also Table 2 for A). Statistically significant differences are indicated by asterisks (∗*p* < 0.05; ∗∗*p* < 0.005; ns, not significant).

For each nanobody, we then compared the ability of the corresponding pair of chimeras (i.e., optimized or non-optimized) to pseudotype VSVΔG-GFP. For this, HEK293T cells were infected by the viruses pseudotyped with the chimeras. For 9 of 11 nanobodies (including C11 and the 8 nanobodies targeting HER2), the infectivity in HEK293T cells of pseudotype production was enhanced when the nanobody was inserted in the optimized backbone. The enhancement factor varied from 6.5 to 29 depending on the nanobody. The geometric mean of the enhancement for these nine nanobodies was 11 (Figure 3A; Table 2). Remarkably, for two chimeras (G_H3_ and G_H4_), the optimized backbone led to a pseudotyping efficiency that was indistinguishable from that of G_WT_ (Table 2). On the other hand, for two others (G_8G5_ and G_9C2_), the optimized backbone had no significant effect on the pseudotyping efficiency (Figure 3A; Table 2).

**Table 2:** Optimization factor for each chimera (indicated by its fused nanobody) in the optimized G backbone context vs. the non-optimized G context in HEK293T cells

Nanobody	log_10_(optimization factor) ± SEM	Optimization factor	log_10_(titer of optimized pseudotype)	Target	*n*	Statistical significance
C11	0.85 ± 0.35	7	6.95 ± 0.35	mCherry	16	∗∗
C8	1.47 ± 0.21	29.5	6.34 ± 0.3	HER2	4	∗∗∗∗
A10	1.26 ± 0.21	18	6.08 ± 0.27	HER2	3	∗∗∗∗
H1	1.05 ± 0.27	11	6.95 ± 0.16	HER2	4	∗∗
H2	1.08 ± 0.38	12	6.75 ± 0.19	HER2	5	∗∗∗
H3	0.81 ± 0.31	6.5	7.24 ± 0.48	HER2	4	ns
H4	0.83 ± 0.22	7	7.23 ± 0.48	HER2	4	ns
H5	1.07 ± 0.21	11.5	6.59 ± 0.23	HER2	5	∗∗∗∗
H6	0.94 ± 0.69	9	6.71 ± 0.46	HER2	3	∗∗
8G5	0.26 ± 0.1	1.8	4.94 ± 0.31	CEACAM	3	∗∗∗∗
9C2	0.17 ± 0.42	1.5	5.18 ± 0.43	Muc1	3	∗∗∗∗

The optimization factor is calculated as the ratio between the titer of VSVΔG-GFP pseudotyped with the optimized chimera and the titer of VSVΔG-GFP pseudotyped with the non-optimized chimera.

The titer of VSVΔG-GFP pseudotyped with the optimized chimera is also indicated, as well as the nanobody target and the number of experiments performed (*n*).

The log_10_ of the titer of VSVΔG-GFP pseudotyped with G_WT_ (used as a reference in each experiment) was 7.3 ± 0.25 (*n* = 16). The statistical significance of the difference between the titer of VSVΔG-GFP pseudotyped with the optimized chimera and the titer of VSVΔG-GFP pseudotyped with G_WT_ is also indicated (∗∗*p* < 0.005; ∗∗∗*p* < 0.0005; ∗∗∗∗*p* < 0.00005; ns, not significant).

### Retargeting of pseudotyped VSVΔG-GFP toward cells expressing HER2 receptor

We showed that the optimization mutations that we had selected with the G_C11_ chimera also improved most of the chimeras involving other nanobodies. However, all of these chimeras remain capable of recognizing the natural VSV receptor (LDL-R).

We therefore introduced the mutations K47Q or R354Q, abolishing receptor recognition by VSV-G,^21^ in the optimized chimera in which VSV-G is fused with nanobodies directed against HER2. We chose to work with chimeras involving nanobodies H1–H4 because these chimeras had the best infectious titers in their optimized version (Table 2). Accordingly, we thus generated the following constructs: G_H1-opt-K47Q_, G_H1-opt-R354Q_, G_H2-opt-K47Q_, G_H2-opt-R354Q_, G_H3-opt-K47Q_, G_H3-opt-R354Q_, G_H4-opt-K47Q_, and G_H4-opt-R354Q_.

As HER2 was expressed at the surface of HEK293T (Figures 3B and 3C), we constructed a HEK293T-HER2^KO^ cell line in which the *ERBB2* gene (encoding HER2 protein) was knocked out (Figures 3B and 3C). We compared the infectivity of VSVΔG-GFP pseudotyped with G_WT_, G mutants incapable of recognizing LDL-R (G_K47Q_ and G_R354Q_), and the different chimeras involving nanobodies H1–H4 (capable or not of recognizing LDL-R). As expected, the titer of VSVΔG-GFP pseudotyped with G_K47Q_ and G_R354Q_ was reduced by a factor of ∼25 (1.4 log_10_) in both cell lines compared to G_WT_ (Figures 3D and 3E). The optimized chimeric glycoproteins G_H1-opt_, G_H2-opt_, G_H3-opt_, and G_H4-opt_, still able to recognize LDL-R, efficiently pseudotyped VSVΔG-GFP in both cell lines. Finally, the titer of VSVΔG-GFP pseudotyped with G_H1-opt-K47Q_, G_H1-opt-R354Q_, G_H2-opt-K47Q_, G_H2-opt-R354Q_, G_H3-opt-K47Q_, G_H3-opt-R354Q_, G_H4-opt-K47Q_, or G_H4-opt-R354Q_ was only slightly reduced in HEK293T cells compared to G_WT_ and similar to G_K47Q_ and G_R354Q_ in HEK293T-HER2^KO^ cells. This indicated that entry in HEK293T cells of VSVΔG-GFP pseudotyped with the different chimeras involving nanobodies H1–H4 and unable to recognize LDL-R is mediated by HER2 (Figures 3D and 3E).

### Chimeras are also functional to retarget lentiviral vectors

**Figure 4:**
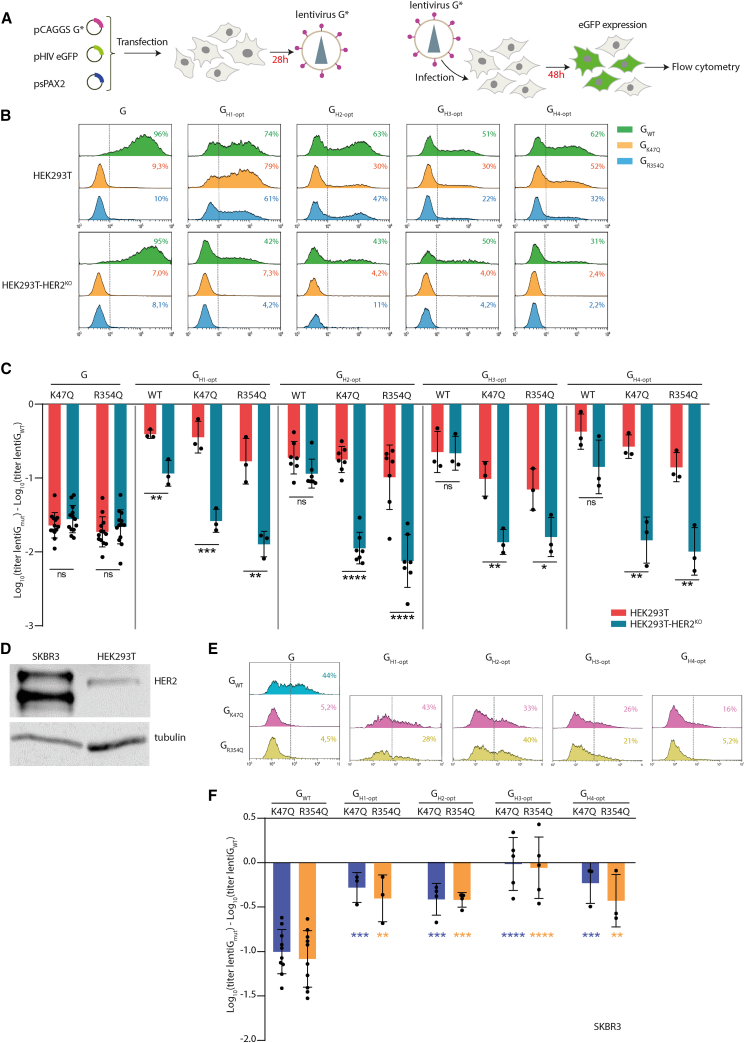
Infectivity of lentiviruses pseudotyped with G_K47Q_, G_R354Q_, or by optimized G chimeras fused to nanobodies H1–H4 in which mutations K47Q or R354Q have been introduced or not (A) Schematic of the experimental design to generate lentivirus pseudotyped by G chimeras (G^∗^) and to measure their infectivity. (B) Flow cytometry graph showing the transduction efficiency in HEK293T and HEK293T-HER2^KO^ cells of lentiviruses pseudotyped with G_WT_, G_K47Q_, G_R354Q_, or with optimized G chimeras fused to nanobodies H1–H4 (recognizing HER2) in which mutations K47Q or R354Q have been introduced or not. The percentage of transduced cells was measured by counting GFP-expressing cells by flow cytometry and indicated on the top right of each graph. (C) Histogram showing the log_10_ of the titer of lentiviruses pseudotyped with the indicated G construct normalized with the one pseudotyped with G_WT_. Each point corresponds to a single experiment as in (B). A value of 0 means that the titer of the lentivirus pseudotyped with mutated or chimeric Gs is equal to the titer of the virus pseudotyped with G_WT_ (in the same cell line). Lentiviral titers were derived from the percentage of cells expressing GFP. The log_10_ of the titer of lentiviruses pseudotyped with G_WT_ was 6.89 ± 0.22 in HEK293T and 6.83 ± 0.22 (*n* = 13) in HEK293T-HER2^KO^ cells. (D) Comparison of the expression of HER2 in HEK293T and SKBR3 cells. Cell lysates were analyzed by western blot with anti-HER2 and anti-tubulin antibodies. The difference of migration of HER2 observed in SKBR3 is likely due to alternative splicing, which is common in cancer cell lines.^42^^,^^43^ (E) Flow cytometry graph showing the transduction efficiency in SKBR3 cells of lentiviruses pseudotyped with G_WT_, G_K47Q_, G_R354Q_, or with optimized G chimeras fused to nanobodies H1–H4 (recognizing HER2) in which mutations K47Q or R354Q have been introduced. The percentage of transduced cells was measured by counting GFP-expressing cells by flow cytometry and indicated on the top right of each graph. (F) Histogram showing the log_10_ of the titer of lentiviruses pseudotyped with the indicated G construct normalized with the one pseudotyped with G_WT_. Each point corresponds to a single experiment as in (E). A value of 0 means that the titer of the lentivirus pseudotyped with mutated or chimeric Gs is equal to the titer of the virus pseudotyped with G_WT_. Lentiviral titers were derived from the percentage of cells expressing GFP 48 h after transduction. The log_10_ of the titer of lentiviruses pseudotyped with G_WT_ was 5.8 ± 0.15 (*n* = 10) in SKBR3 cells. In (C) and (F), error bars represent the SD for experiments carried out at least in triplicate. Statistically significant differences are indicated by asterisks (∗*p* < 0.05; ∗∗*p* < 0.005; ∗∗∗*p* < 0.0005; ∗∗∗∗*p* < 0.00005; ns, not significant). In (F), blue (resp. orange) asterisks indicate the significance of the difference between G_K47Q_ (resp. G_R354Q_) and the chimera having the same mutation.

VSV-G is the most used viral glycoprotein to pseudotype lentiviral vectors (Figure 4A). We decided to investigate if the chimera in which VSV-G, carrying the mutation K47Q or R354Q, is fused with nanobodies directed against HER2 could specifically redirect lentiviruses toward HER2-expressing cells. To do this, we compared the infectivity of GFP-encoding lentiviruses pseudotyped with these different chimeras in HEK293T and HEK293T-HER2^KO^. Infectivity was analyzed by counting GFP-expressing cells using flow cytometry (Figure 4A).

The results indicated that lentivirus pseudotyped with chimeras G_H1-opt-K47Q_, G_H1-opt-R354Q_, G_H2-opt-K47Q_, G_H2-opt-R354Q_, G_H3-opt-K47Q_, G_H3-opt-R354Q_, G_H4-opt-K47Q_, or G_H4-opt-R354Q_ infected much more efficiently HEK293T that HEK293T-HER2^KO^ (Figures 4B and 4C). This indicated that the lentiviruses were retargeted against cells that express HER2.

This was confirmed using SKBR3 cells that express high level of HER2 at their surface (Figure 4D). Lentiviruses pseudotyped with chimeras G_H1-opt-K47Q_, G_H1-opt-R354Q_, G_H2-opt-K47Q_, G_H2-opt-R354Q_, G_H3-opt-K47Q_, G_H3-opt-R354Q_, G_H4-opt-K47Q_, or G_H4-opt-R354Q_ infected more efficiently this cell line than those pseudotyped with G_K47Q_ or G_R354Q_ (Figures 4E and 4F).

### Chimeras specifically target HER2-expressing cells

**Figure 5:**
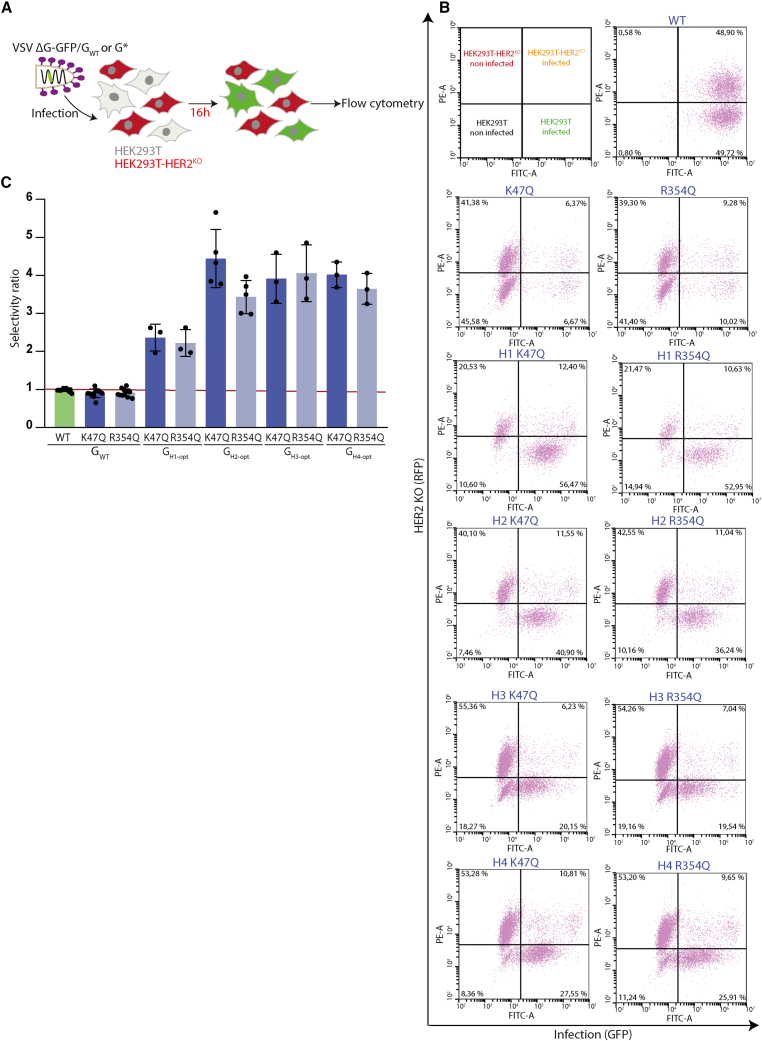
VSV pseudotyped with optimized G_K47Q_ or G_R354Q_ chimeras fused to nanobodies H1–H4 preferentially infect HEK293T WT cells (A) Schematic of the experimental design: A 1:1 mix of HEK293T and HEK293T-HER2^KO^ (the latter expressing the RFP protein) was infected with VSVΔG-GFP pseudotyped with the indicated G construct. (B) Representative biparametric flow cytometry graph showing the infectivity of VSVΔG-GFP pseudotyped with the indicated glycoprotein in each cell population (*x* axis: intensity of GFP fluorescence *i.e.* infection; *y* axis: intensity of RFP *i.e.* cell line identity). (C) Selectivity index of VSVΔG-GFP pseudotyped with the chimeras. The selectivity index was calculated by dividing the proportion of infected HEK293T cells by the proportion of infected HEKT293-HER2^KO^ cells in the mix. Each data point corresponds to a single experiment. For each G construct, the mean of experiments carried out at least in triplicate is indicated. The error bars represent the SD.

We then assessed the specificity of the viral retargeting. For this, we co-cultured HEK293T and HEK293T-HER2^KO^ (expressing RFP) cells. Each cell line constituted about half of the total cell population at the time of infection by VSVΔG-GFP pseudotyped with different forms of G (Figure 5A). As expected, VSVΔG-GFP pseudotyped with G_WT_ infected HEK293T and HEK293T-HER2^KO^ cell lines equally efficiently, whereas those pseudotyped with G_K47Q_ and G_R354Q_ were virtually unable to infect either cell line (Figure 5B). Interestingly, confirming our previous results, VSVΔG-GFP pseudotyped with G_H1-opt-K47Q_, G_H1-opt-R354Q_, G_H2-opt-K47Q_, G_H2-opt-R354Q_, G_H3-opt-K47Q_, G_H3-opt-R354Q_, G_H4-opt-K47Q_, or G_H4-opt-R354Q_ selectively infected HEK293T cells in the mixed population. A selectivity index was calculated by dividing the proportion of infected HEK293T cells by the proportion of infected HEK293T-HER2^KO^ cells. The selectivity indexes of the VSVΔG-GFP pseudotyped with the chimeras range from ∼2.25 for chimeras involving H1 to ∼4 for the other nanobodies (H2, H3, and H4) (Figure 5C).

## Discussion

One of the major challenges of *in vivo* therapy, including oncovirotherapy and gene therapy, is the ability to target specifically cells of interest. Indeed, the lack of specificity can lead not only to off-target toxicity but also, in the case of gene therapy (particularly chimeric antigen receptor therapies), to trapping of the vector in non-targeted cells. The identification of mutations in VSV-G that abolish LDL-R recognition without affecting fusion activity has opened the possibility of specifically targeting cells of interest.^21^

Indeed, these VSV-G mutants have been used in combination with a second TM protein, which binds a ligand/receptor of interest, to pseudotype lentiviruses, and specifically target them toward a given cell type.^28^^,^^29^^,^^30^^,^^31^ As second TM protein, a dimerized, surface-tethered interleukin (IL)-13 allowed a specific infection of IL-13Rα1-expressing cells,^30^ while a TM version of an anti-CD19 single-chain antibody fragment (scFv) efficiently infected CD19^+^ B cell lines, but not CD19^-^ T cell lines,^30^ as well as CD19-expressing HEK293T, but not the parental HEK293T cell line.^28^

Recently, a gene encoding VSV-G carrying mutations K47Q and R354Q, fused to a sequence coding for an scFv directed against HER2, was introduced in place of the VSV-G_WT_ gene into the genome of a recombinant VSV. This virus was then adapted through serial passages on HER2-expressing mouse mammary tumor line. The resulting virus has a 15- to 25-fold higher titer in HER2-expressing cell lines than in HER2-negative cells.^32^ Adaptive mutations occurred in both VSV-G and scFv and, therefore, are specific to the scFv used in the study.

Here, using experimental evolution, we selected two mutations that optimize the VSV-G backbone to tolerate an amino-terminal fusion with a nanobody recognizing mCherry. The choice of this nanobody had the advantage of limiting a possible GOF of the nanobody. Indeed, performing experimental evolution on a chimera involving a nanobody recognizing a cellular receptor could have resulted in mutations leading to increased affinity for its ligand (without optimizing G backbone, which was our objective). Interestingly, the selected mutations also improved the functionality of chimeric constructs involving other HER2-directed nanobodies from several libraries. When used to pseudotype VSVΔG-GFP, the optimized chimeric constructs showed an ∼10-fold improvement over the non-optimized chimera. This is particularly promising given the large number of nanobody libraries available, potentially enabling the targeting of virtually any receptor of interest provided it can mediate endocytosis of the viral particle so that it ends up in an acidic environment propitious to VSV-G-mediated fusion.

Remarkably, the S422I mutation emerged in another context to compensate for the H407A mutation, which abolished the fusion properties of G.^20^ Structural analysis showed that this mutation stabilizes a β-hairpin motif through hydrophobic stacking of residues I422 between F424 and L430.^20^ Here, we show that introduction of other hydrophobic residues at position 422 has the same chimera-optimizing effect, suggesting that stabilization of this structural motif is critical in the context of N-terminal fusions. On the other hand, we do not have any molecular explanation for the selection of mutation H22N. Indeed, structural prediction using AlphaFold 3^33^ revealed no detectable impact of this mutation on the structure of VSV-G or G_C11_. It should also be noted that to identify histidines that could act as pH-sensitive switches, we previously constructed and characterized the H22A mutant, which was shown to have no impact on the phenotype of VSV-G.^20^

Last but not least, after introducing the K47Q and R354Q mutations in the context of optimized chimeras with four nanobodies recognizing HER2, we established the proof of concept that it is possible to specifically redirect the infection of pseudotyped VSV or pseudotyped lentiviruses toward cells expressing this receptor. Our selectivity test, performed using mixed population of HER2-positive and -negative cells, showed the capacity of retargeted viruses to specifically infect the subpopulation of positive cells. This specificity is 4-fold, which might appear modest. However, HEK293T cells do not express much of this receptor on their surface; the specificity could be much greater in the context of a tumor within which cancer cells can express very high levels of this receptor.^26^

For retargeting, the bipartite approach (i.e., using a second membrane protein binding the receptor of interest)^28^^,^^29^^,^^30^^,^^31^ or the chimeric approach that we have developed here gives similar results in terms of specificity. Nevertheless, being able to combine receptor recognition and fusion activity on a single chimeric protein is an advantage for having homogeneous pseudotyped viruses. In contrast, in the bipartite approach, the ratio between the two proteins on the surface of the viruses varies from one particle to another, potentially affecting reproducibility and infectivity. Finally, for the development of replicative oncolytic viruses, the chimeric approach is optimal in terms of the quantity of genetic material inserted in the viral genome as it requires the insertion of only one gene into the viral genome.

Overall, the introduction of the two mutations H22N and S442I (or any other hydrophobic aa in position 422) will therefore make it possible to have functional chimeric glycoproteins suitable for pseudotyping both VSV and lentiviruses, or even for constructing replicative recombinant VSVs, targeting any receptor of interest through the nanobody. Furthermore, these chimeras can be incorporated into the membrane of extracellular vesicles enabling targeted delivery of their cargo into cells of interest for therapeutic purpose.^34^^,^^35^ Of note, it is known that VSV-G pseudotyped lentiviruses are poorly effective in infecting certain cell types that poorly express LDL-R on their surface.^36^ The strategy described here may thus also be used in the context of *ex vivo* gene therapy using a mutant chimeric G targeting a receptor of interest different from the LDL-R.

## Material and methods

### Cell lines

BSR, a clone of BHK-21 (baby hamster kidney; ATCC CCL-10), and HEK293T (human embryonic kidney expressing simian virus 40 T antigen [SV40T]; ATCC CRL-3216) cells were grown in DMEM supplemented with 10% fetal calf serum (FCS). SKBR3 (human breast cancer cell line; ATCC HTB-30) cells were grown in McCoy’s 5A (modified) medium supplemented with 10% FCS.

### Antibodies

Mouse mAb directed against VSV-G ectodomain (8G5F11; Kerafast) was used for flow cytometry at a dilution of 1:1,500. Another mouse anti-VSV-G antibody (SAB4200695; Sigma-Aldrich) was used for western blot analysis at a 1:5,000 dilution. Mouse mAb 24A1 (kindly provided by Robin Weiss) was used to detect VSV nucleoprotein by immunofluorescence. A mouse anti-HER2 antibody (MA512759; Invitrogen) was used for flow cytometry at a 1:200 dilution. Another mouse anti-HER2 antibody (MA513105; Invitrogen) was used in western blot at a 1:200 dilution. Goat anti-mouse Alexa Fluor 488 (10696113; Invitrogen) was used as secondary antibody for flow cytometry (1:1,000 dilution). Goat anti-mouse IgG conjugated to peroxidase (A4416; Merck) was used as the secondary antibody at a dilution of 1:5,000 in western blot analysis.

### Plasmids and cloning

The cDNAs encoding the nanobodies were ordered from Eurofins Genomics or Twist Bioscience. For anti-HER2 nanobodies, sequences were retrieved from the NCBI database (H1: MP600335, H2: MP600344, H3: MP600340, H4: MP600341, H5: MP600327, H6: MP600331). Sequences of nanobodies C11, C8, A10, 8G5, and 9C2, all derived from the same library,^25^ are available upon request. Chimeric glycoproteins and point mutations were generated starting from VSV-G gene (Indiana Mudd-Summer strain) cloned in the pCAGGS plasmid (NovoPro).^20^ For this, overlapping DNA fragments were amplified by PCR and assembled with the linearized plasmid using the Gibson Assembly kit (New England Biolabs) following the manufacturer’s instructions.

### CRISPR-Cas9-mediated *ERBB2* gene knockout

*ERBB2* gene (encoding the HER2 protein) was knocked out in HEK293T cells using CRISPR-Cas9 knockout (KO) and HDR (homologous-directed recombination) plasmids from Santa Cruz Biotechnology.^37^ Briefly, HEK293T were cells transfected with 1 μg of a pool of three plasmids (CRISPR/Cas9 KO ErbB2/HER2 (h2) (sc-400138-KO-2)) each encoding the Cas9 nuclease but distinct ERBB2-specific guide RNA and 1 μg of HDR plasmid (ErbB2/HER2 (h2) (sc-400138-HDR-2)), also encoding RFP, were co-transfected for selection. HEK293T-HER2^KO^ cells were then selected using 0.75 μg/mL of puromycin. Successful KO of ERBB2 was confirmed by flow cytometry and western blot analysis. As a result of the experimental procedure, HEK293T-HER2^KO^ cells also express RFP.

### Cell surface expression

To quantify cell surface expression of VSV-G, HEK293T cells plated in 6-well dishes at 70% confluence were transfected with G-expressing plasmid using the calcium phosphate method. At 24 h after transfection, cells were detached using 1 mL of PBS (10 mM Na_2_HPO_4_, 1.8 mM KH_2_PO_4_, 137 mM NaCl, 2.7 mM KCl, pH 7.4) containing 2% FBS (fetal bovine serum). A 200 μL aliquot of the cell suspension was transferred to a 96-well plate and centrifuged at 350 g for 3 min. Cells were then incubated with a 1:1,500 dilution of mouse monoclonal anti-G antibody (8G5F11) in PBS at 4°C for 30 min. Cells were washed twice with PBS, incubated with a 1:1,000 dilution of goat anti-mouse Alexa Fluor 488 (Invitrogen) at 4°C for 30 min, and rinsed in PBS. After resuspension in 100 μL of 2% FBS-PBS, the fluorescence of 10,000 cells from each population was determined by flow cytometry using a CytoFLEX S flow cytometer. The median fluorescence intensity (MFI) of the transfected cells expressing G was quantified by flow cytometry. The relative cell surface expression of transfected cells was calculated as the ratio of MFI for each G variant to the MFI of G_WT_ (mutant MFI/G_WT_ MFI). For each mutant, the percentage given is the average of at least three independent experiments.

### Generation of rVSV-G_C11_ recombinant

Plasmid pVSV-FL(+) expressing the 11,161-nucleotide positive-strand full-length VSV RNA sequence and plasmids pBS-N, pBS-P, and pBS-L, respectively, encoding N, P, and L proteins were kindly provided by John K. Rose (Yale University, New Haven, CT, USA). The chimeric G gene from the VSV Indiana serotype (Mudd-Summers strain) in fusion with that encoding the nanobody C11 was inserted into the original full-length genomic plasmid pVSV-FL(+)^38^ using two unique restriction sites: MluI, located in the 5′ noncoding region of the G gene, and NheI, located in an inserted sequence between the G and L genes, after removal of the corresponding VSV Indiana Mudd-Summers G gene. Recombinant rVSV-G_C11_ was recovered in BSR cells as previously described.^39^ Briefly, BSR cells were infected with recombinant vaccinia virus vTF7-3 expressing the bacteriophage T7 RNA polymerase at an MOI of 10. After 1 h, BSR cells were co-transfected with the pVSV-FL(+), pBS-N, pBS-P, and pBS-L plasmids using Lipofectamine 2000 (Invitrogen), in the presence of 10 μg/mL 1-β-d-arabinofuranosylcytosine (araC; Sigma) to inhibit vaccinia virus replication. The recovery of the recombinant virus was supported by *trans*-complementation with VSV G_WT_,^20^^,^^40^^,^^41^ provided from a plasmid (pcDNA3.1 G_WT_) that was cotransfected with plasmids pVSV-FL(+), pBS-N, pBS-P, and pBS-L.

### Experimental evolution of rVSV-G_C11_ and sequencing

Experimental evolution rVSV-G_C11_ was achieved by serial passaging of the virus at a very low MOI (∼3x10^−4^) on BSR cells. At each passage, the virus was titrated by plaque assay. Infected cells were lysed 6 h post-infection in RNAnow reagent (Ozyme). Total RNA was extracted, mRNA was reverse transcribed using a T_12_CAT primer, and the cDNA corresponding to M and G mRNAs were amplified by PCR. The PCR products were sequenced by the Sanger method (Eurofins).

### Virus purification

rVSV-G_C11_ was propagated on BSR cells. Sixteen hours post-infection, cell debris were eliminated from the supernatant by filtration through a 0.22-μm filter. The virus was then pelleted by centrifugation at 4°C for 45 min at 25,000 rpm using an SW 28 rotor (Beckman). The viral pellet was gently resuspended in dilution buffer (150 mM NaCl, 10 mM Tris-HCl pH 7.5, 2 mM EDTA).

### Preparation of VSVΔG-GFP pseudotypes

HEK293T cells at 80% confluence in 6-well dishes were transfected with 1 μg of pCAGGS plasmids encoding WT or chimeric Gs using the calcium phosphate method. Twenty-four hours after transfection, cells were infected with VSVΔG-GFP pseudotyped with VSV G_WT_ at an MOI of 3. One hour post-infection, 1 mL of complete DMEM (i.e., supplemented with 10% FCS and penicillin/streptomycin) was added. At 2 h post-infection, cells were washed to remove residual viruses from the inoculum, and 2 mL of complete DMEM was added. Cell supernatants containing the newly pseudotyped viral particles were collected at 16 h post-infection and clarified by centrifugation at 600 *g* for 15 min at 4°C to remove cell debris.

### Titration of VSVΔG-GFP pseudotypes

The infectious titers of pseudotyped VSVΔG-GFP were calculated by counting cells expressing the GFP using flow cytometry at 16 h post-infection. The MOI was calculated using the equation MOI = −ln[p(0)], where p(0) is the proportion of non-infected cells. The viral titer was deduced from the MOI, considering the volume and dilution of the viral inoculum as well as the total number of cells.

### Preparation of lentivirus pseudotypes

HEK293T cells at 80% confluence in 6-well dishes were co-transfected using 200 ng of pCAGGS encoding G_WT_ or chimeric Gs, 400 ng of psPAX2 encoding HIV-1 gag and pol (Addgene #12260), and 500 ng of pHIV-eGFP encoding eGFP (Addgene#2137) using polyethylenimine (PEIMax; Polyscience) at 1 mg/mL. At 18 h after transfection, the culture medium was replaced with fresh complete DMEM. After 28 h, cell supernatants containing the pseudotyped viral particles were collected and clarified by centrifugation at 600 *g* for 15 min at 4°C to remove the cell debris.

### Titration of lentivirus pseudotypes

The infectious titers of pseudotyped lentiviruses were calculated by counting cells expressing the GFP using flow cytometry at 48 h post-transduction. The MOI was calculated using the equation MOI = −ln[p(0)], where p(0) is the proportion of non-infected cells. The viral titer was deduced from the MOI, considering the volume and dilution of the viral inoculum as well as the total number of cells.

### Western blot analysis

Supernatant containing VSVΔG-GFP pseudotypes were harvested and centrifuged at 2,500 rpm for 15 min at 4°C. Clarified viral supernatant was concentrated by ultracentrifugation at 40,000 *g* in an SW 55 rotor for 45 min at 12°C. The pellets were resuspended directly in 100 μL of TNE buffer (20 mM Tris-HCl pH 7.5, 50 mM NaCl, 2 mM EDTA); 60 μL of the resuspension was mixed with 20 μL of Laemmli buffer and heated at 95°C for 5 min.

HEK293T, HEK293T-HER2^KO^, and SKBR3 cells were pelleted by centrifugation at 600 × *g* for 10 min at 4°C. Pellets were resuspended in RIPA buffer (Tris-HCl 25 mM pH 8.8, NaCl 50 mM, 0.5% NP-40, 0.1% SDS) supplemented with a cocktail of protease inhibitors (Roche Diagnostic) and incubated on ice for 30 min. Lysates (equivalent to 3x10^4^ cells) were then centrifuged at 9,500 *g* for 2 min, and supernatants were denatured in Laemmli buffer and heated at 95°C for 5 min.

For the western blot analysis, proteins were separated on 12% SDS-PAGE and transferred to a nitrocellulose membrane. Membranes were blocked in TBS-0.1% Tween 20 supplemented with 5% non-fat dry milk (TBS, Tris-buffered saline pH 7.5; Euromedex) for 45 min. The membranes were then incubated overnight at 4°C with the primary antibodies diluted in TBS-0.1% Tween 20 supplemented with 1% non-fat dry milk. After 3 washes, the membrane was incubated with the secondary antibody for 1 h at room temperature. After 2 washes, the membranes were developed using SuperSignal West Pico PLUS reagent form Thermo Fisher Scientific and imaged with an Odyssey infrared imaging system (LI-COR, Lincoln, NE, USA).

### Electron microscopy

Purified virion samples were either diluted in 150 mM NaCl and 50 mM Tris-HCl pH 7.5 or dialyzed against a buffer containing 150 mM NaCl and 50 mM MES (2-(N-morpholino)ethanesulfonic acid) at pH 5.5 and analyzed by conventional transmission electron microscopy using the negative staining method. Three microliters of each sample were applied on an air glow-discharged 400 mesh copper carbon-coated grid for 1 min. The excess of liquid was blotted off, and the grids were stained with sodium phosphotungstic acid adjusted to the corresponding pH (7.5 or 5.5). Imaging was performed at 100 kV using a Tecnai 12 Spirit transmission electron microscope (Thermo Fisher Scientific, New York NY, USA) equipped with a K2 Base 4k × 4k camera (Gatan, Pleasanton CA, USA).

### Statistical analysis

Statistical analysis was performed using Prism software (GraphPad). Individual data points are indicated on the corresponding graphs. Data comparison was performed using unpaired two-tailed Student’s t test. Statistical details of experiments are indicated in the figure legends. Results are indicated as mean ± SD.

## Data and code availability

The data that support the findings of this study are available from the corresponding authors (Y.G. or A.A.A.).

## Acknowledgments

This work was supported by a grant from 10.13039/501100002915Fondation pour la Recherche Médicale (FRM), France, to Y.G. (EQU202103012746); a grant from l’Agence pour la Recherche sur le Cancer (ARC), France, to A.A.A.; and by a grant from la Banque Publique d’Investissement (Projet ETINCELL) including a salary for I.D. The work in F.P. lab was supported by the 10.13039/501100004794CNRS and by the Institut Curie as well as by Labex Cell(n)Scale (ANR-10-LBX-0038 part of the IDEX PSL no. ANR-10- IDEX-0001-02). The present work benefited from the Cryo-EM platform of I2BC supported by the French Infrastructure for Integrated Structural Biology ANR-10-INBS-05.

## Author contributions

I.D. performed most of the experiments and analyzed the data with A.A.A. and Y.G. S.T. and S.J. contributed to the characterization of the infectivity of the pseudotypes. H.R. constructed the recombinant virus and performed experimental evolution. M.O. performed electron microscopy. S.M. and F.P. provided the sequence of some of the nanobodies used in this study. F.P. contributed to many discussions leading up to this project. A.A.A. and Y.G. conceived, designed, and co-supervised the study. A.A.A. and Y.G. wrote the manuscript. C.L.-G. and F.P. revised the manuscript. All authors read and approved the final version of the manuscript.

## Declaration of interests

The mutants described in this work are the subject of a patent application files by the CNRS on which H.R., F.P., A.A.A. and Y.G. are named as inventors.
